# Ending nuclear weapons, before they end us

**DOI:** 10.1111/apa.70138

**Published:** 2025-06-02

**Authors:** Kamran Abbasi, Parveen Ali, Virginia Barbour, Marion Birch, Inga Blum, Peter Doherty, Andy Haines, Ira Helfand, Richard Horton, Kati Juva, Jose F. Lapena, Robert Mash, Olga Mironova, Arun Mitra, Carlos Monteiro, Elena N. Naumova, David Onazi, Tilman Ruff, Peush Sahni, James Tumwine, Carlos Umaña, Paul Yonga, Chris Zielinski

**Affiliations:** ^1^ BMJ; ^2^ International Nursing Review; ^3^ Medical Journal of Australia; ^4^ Medact & University College London; ^5^ IPPNW; ^6^ 1996 Nobel Prize for Physiology or Medicine; ^7^ London School of Hygiene and Tropical Medicine; ^8^ IPPNW; ^9^ The Lancet; ^10^ IPPNW; ^11^ WAME; ^12^ African Journal of Primary Health Care & Family Medicine; ^13^ IPPNW; ^14^ IPPNW; ^15^ Revista de Saúde Pública; ^16^ Journal of Public Health Policy; ^17^ IPPNW; ^18^ IPPNW; ^19^ National Medical Journal of India; ^20^ African Health Sciences; ^21^ IPPNW; ^22^ East African Medical Journal; ^23^ WAME

This May, the World Health Assembly (WHA) will vote on re‐establishing a mandate for the World Health Organization (WHO) to address the health consequences of nuclear weapons and war [1]. Health professionals and their associations should urge their governments to support such a mandate and support the new UN comprehensive study on the effects of nuclear war.

The first atomic bomb exploded in the New Mexico desert 80 years ago, in July 1945. Three weeks later, two relatively small (by today's standards), tactical‐size nuclear weapons unleashed a cataclysm of radioactive incineration on Hiroshima and Nagasaki. By the end of 1945, about 213,000 people were dead [2]. Tens of thousands more have died from late effects of the bombings.

Last December, Nihon Hidankyo, a movement that brings together atomic bomb survivors, was awarded the Nobel Peace Prize for its “efforts to achieve a world free of nuclear weapons and for demonstrating through witness testimony that nuclear weapons must never be used again” [3]. For the Norwegian Nobel Committee, the award validated the most fundamental human right: the right to live. The Committee warned that the menace of nuclear weapons is now more urgent than ever before. In the words of Committee Chair Jørgen Watne Frydnes, “it is naive to believe our civilisation can survive a world order in which global security depends on nuclear weapons. The world is not meant to be a prison in which we await collective annihilation.” [4] He noted that our survival depended on keeping intact the “nuclear taboo” (which stigmatises the use of nuclear weapons as morally unacceptable) [5].

The nuclear taboo gains strength from recognition of compelling evidence of the catastrophic humanitarian consequences of nuclear war, its severe global climatic and famine consequences, and the impossibility of any effective humanitarian response. This evidence contributed significantly to ending the Cold War nuclear arms race [6, 7].

While the numbers of nuclear weapons are down to 12,331 now, from their 1986 peak of 70,300 [8], this is still equivalent to 146,605 Hiroshima bombs [9], and does not mean humanity is any safer [10]. Even a fraction of the current arsenal could decimate the biosphere in a severe mass extinction event. The global climate disruption caused by the smoke pouring from cities ignited by just 2% of the current arsenal could result in over two billion people starving [11].

A worldwide nuclear arms race is underway. Deployed nuclear weapons are increasing again, and China, India, North Korea, Pakistan, Russia and UK are all enlarging their arsenals. An estimated 2,100 nuclear warheads in France, Russia, UK, US and, for the first time, also in China, are on high alert, ready for launch within minutes [8]. With disarmament in reverse, extensive nuclear modernisations underway, multiple arms control treaties abrogated without replacement, no disarmament negotiations in evidence, nuclear‐armed Russia and Israel engaged in active wars involving repeated nuclear threats, Russia and the US deploying nuclear weapons to additional states, and widespread use of cyberwarfare, the risk of nuclear war is widely assessed to be greater than ever. This year the Doomsday Clock was moved the closest to midnight since the Clock's founding in 1947 [10].

Led by Ireland and New Zealand, in late 2024, the United Nations General Assembly (UNGA) voted overwhelmingly to establish a 21‐member independent scientific panel to undertake a new comprehensive study on the effects of nuclear war [12], with its final report due in 2027. Noting that “removing the threat of a nuclear war is the most acute and urgent task of the present day”, the panel has been tasked with examining the physical effects and societal consequences of a nuclear war on a local, regional and planetary scale. It will examine the climatic, environmental and radiological effects of nuclear war, and their impact on public health, global socioeconomic systems, agriculture and ecosystems.

The resolution calls upon UN agencies, including WHO, to support the panel's work, including by “contributing expertise, commissioned studies, data and papers”. All UN Member States are encouraged to provide relevant information, scientific data and analyses; facilitate and host panel meetings, including regional meetings; and make budgetary or in‐kind contributions. Such an authoritative international assessment of evidence on the most acute existential threat to humankind and planetary health is long overdue. The last such report dates from 1989. It is shameful that France, UK and Russia opposed this resolution [13].

In 1983 and 1987 [14], WHO convened an international committee of scientists and health experts to study the health effects of nuclear war. Its landmark, authoritative reports were influential and an excellent example of WHO fulfilling its constitutional mandate “to act as the directing and coordinating authority on international health work”. In 1993, WHO produced an additional shorter report on the health and environmental effects of nuclear weapons, which included discussion of the production chain of nuclear weapons, including processing, testing and disposal [15].

However, despite WHA having mandated WHO to report periodically on relevant developments, no further work was undertaken and in 2020 WHO's mandate on nuclear weapons and health lapsed.

The Marshall Islands, Samoa and Vanuatu, supported by seven co‐sponsoring states and International Physicians for the Prevention of Nuclear War (IPPNW), are working to renew WHO's mandate. They are seeking wide support for a resolution on the health effects of nuclear weapons/war at this year's WHA in Geneva on 19‐27 May [1]. WHO would then re‐establish a programme of work on this most critical threat to health, and be able to lead strongly in providing the best health evidence to the UN panel.

Health professionals are well aware how crucial accurate and up‐to‐date evidence is to making good decisions. We and our organisations should support such a renewed mandate by urging our national WHA delegates to vote in support and commit the modest funds needed to re‐establish WHO's work programme, especially now, as the organisation faces severe financial strain with the US decision to withdraw its membership.

Our joint editorial in 2023 [16] on reducing the risks of nuclear war and the role of health professionals, published in over 150 health journals worldwide, urged three immediate steps by nuclear‐armed states and their allies: adopt a “no first use” policy, take their nuclear weapons off hair‐trigger alert, and pledge unequivocally that they will not use nuclear weapons in any current conflicts they are involved in. We also urged nuclear‐armed states to work for a definitive end to the nuclear threat by urgently starting negotiations for a verifiable, timebound agreement to eliminate their nuclear arsenals, and called on all nations to join the Treaty on the Prohibition of Nuclear Weapons [17].

It is an alarming failure of leadership that no progress has been made on these needed measures, nor on many other feasible steps away from the brink, acting on the obligation of all states to achieve nuclear disarmament. Nine states jeopardise all humanity and the biosphere by claiming an exclusive right to wield the most destructive and inhumane weapons ever created. The world desperately needs the leaders of these states to freeze their arsenals, end the modernisation and development of new, more dangerous nuclear weapons, and ensure that new technology such as artificial intelligence can never trigger the launch of nuclear weapons.

The UN scientific panel and a renewed mandate for WHO's work in this area can provide vital authoritative and up‐to‐date evidence for health and public education, evidence‐based advocacy and policies, and the mobilised public concern needed to trigger decisive political leadership. This is a core health imperative for all of us.

## References

[1] World Health Organization . Effects of nuclear weapons and war on health and health services. EB156/CONF./10, Executive Board, 5 Feb 2025. https://apps.who.int/gb/ebwha/pdf_files/EB156/B156_CONF10‐en.pdf (accessed 4/3/2025)

[2] M. Tomonaga , “The atomic bombings of Hiroshima and Nagasaki: a summary of the human consequences, 1945‐2018, and lessons for *Homo sapiens* to end the nuclear weapon age,” Journal for Peace and Nuclear Disarmament 2, no. 2 (2019): 491–517.

[3] NobelPrize.org , Nobel Peace Prize (2024): 2024, https://www.nobelprize.org/prizes/peace/2024/summary/ accessed 25/2/2025.

[4] Award ceremony speech . NobelPrize.org. Nobel Prize Outreach 2025. Tue. 25 Feb 2025. https://www.nobelprize.org/prizes/peace/2024/ceremony‐speech/ (accessed 25/2/2025)

[5] Tannenwald, Nina . The Nuclear Taboo: The United States and the Normative Basis of Nuclear Non‐Use. International Organization, vol. 53, no. 3, 1999, pp. 433–468. *JSTOR*, http://www.jstor.org/stable/2601286 (accessed 3/3/2025)

[6] A. Robock , L. Xia , C. S. Harrison , J. Coupe , O. B. Toon , and C. Bardeen , “Opinion: How fear of nuclear winter has helped save the world, so far,” Atmospheric Chemistry and Physics 23, no. 12 (2023): 6691–6701, https://acp.copernicus.org/articles/23/6691/2023/ accessed 4/3/2025.

[7] I. Helfand , A. Haines , T. Ruff , H. Kristensen , P. Lewis , and Z. Mian , “The growing threat of nuclear war and the role of the health community,” World Medical Journal 62, no. 3 (2016): 86–94.

[8] H. Kristensen , M. Korda , E. Johns , M. Knight , and K. Kohn , “Status of world nuclear forces,” Federation of American Scientists., https://fas.org/initiative/status‐world‐nuclear‐forces/ accessed 18/3/2025.

[9] Norwegian People's Aid . Nuclear weapons ban monitor 2024. February 2025. https://banmonitor.org/ (accessed 25/3/2025)

[10] Science and Security Board . Closer than ever: It is now 89 seconds to midnight. 2025 Doomsday Clock Statement. Bulletin of the Atomic Scientists. 28 Jan 2025. https://thebulletin.org/doomsday‐clock/2025‐statement/ (accessed 4/3/2025)

[11] L. Xia , A. Robock , K. Scherrer , et al., “Global food insecurity and famine from reduced crop, marine fishery and livestock production due to climate disruption from nuclear war soot injection,” Nat Food 3 (2022): 586–596. 37118594 10.1038/s43016-022-00573-0

[12] United Nations General Assembly. Nuclear war and scientific research. A/C.1/79/L.39 15 Oct 2024. https://reachingcriticalwill.org/images/documents/Disarmament‐fora/1com/1com24/resolutions/L39‐.pdf (accessed 4/3/2025)

[13] https://reachingcriticalwill.org/images/documents/Disarmament‐fora/1com/1com24/votes‐ga/408DRXVII.pdf (accessed 4/3/25)

[14] World Health Organization , Effects of nuclear war on health and health services, 2nd ed. (WHO, 1987), https://iris.who.int/handle/10665/39199 accessed 4/3/2025.

[15] World Health Organization . Health and environmental effects of nuclear weapons. WHA46/30. 26 April 1993. https://iris.who.int/bitstream/handle/10665/175987/WHA46_30_eng.pdf?isAllowed=y&sequence=1 (accessed 4.3.2025)

[16] K. Abbasi , P. Ali , V. Barbour , et al., “Reducing the risks of nuclear war,” BMJ 382 (2023): 1682. 37527852 10.1136/bmj.p1682

[17] United Nations , Treaty on the Prohibition of Nuclear Weapons 2017 https://www.icanw.org/tpnw_full_text accessed 9/3/2025.

